# Amplifying Cognitive Functions in Amateur Esports Athletes: The Impact of Short-Term Virtual Reality Training on Reaction Time, Motor Time, and Eye–Hand Coordination

**DOI:** 10.3390/brainsci14111104

**Published:** 2024-10-30

**Authors:** Maciej Lachowicz, Anna Serweta-Pawlik, Alicja Konopka-Lachowicz, Dariusz Jamro, Grzegorz Żurek

**Affiliations:** 1 Department of Biostructure, Wroclaw University of Health and Sport Sciences, 51-612 Wrocław, Poland; grzegorz.zurek@awf.wroc.pl; 2 Department of Occupational Therapy, Wroclaw University of Health and Sport Sciences, 51-612 Wrocław, Poland; anna.serweta@awf.wroc.pl; 3 Fizjohome Rehabilitation, 53-201 Wrocław, Poland; akonopka011@gmail.com; 4 Department of Physical Education and Sport, General Tadeusz Kosciuszko Military University of Land Forces, 51-150 Wrocław, Poland; dariusz.jamro@awl.edu.pl

**Keywords:** cognitive function, esports, reaction time, eye–hand coordination, virtual reality

## Abstract

Objectives: Electronic sports (esports) have grown into a major competitive field in today’s digital landscape, attracting the interest of established companies and evolving into a fast-growing industry. Cognitive function, including reaction time, motor time, and eye–hand coordination, plays a crucial role in e-athlete performance. This study aims to examine the impact of VR training on these cognitive functions in amateur e-athletes. Methods: The study involved 66 amateur e-athletes (45 men and 21 women, aged 19–41, with a mean age of 23.96 ± 3.90 years) who reported active, non-professional involvement in esports. Participants were randomly assigned to an experimental group (E) (n = 32) and a control group (C) (n = 34), with initial comparisons confirming no significant differences in daily gaming habits, esports experience, or age between groups. The E group completed 15-minute daily training sessions using the VR game Beat Saber over eight consecutive days. Results: The results demonstrated that VR training significantly improved eye–hand coordination in the experimental group, although there were no notable effects on reaction time or motor time. Conclusions: These findings suggest that VR training may be an effective method to enhance certain cognitive functions, specifically eye–hand coordination, among amateur e-athletes. This could offer a valuable approach for performance improvement in this rapidly growing field.

## 1. Introduction

Sport has been integral to culture since the inaugural Olympic Games in 776 BC, evolving to meet changing societal interests [1,2]. Today, sports are cultural and economic forces, supported by global organizations and modern technology [3,4]. Emerging sports like parkour and drone racing offer new forms of competition, challenging traditional norms and appealing to those seeking novel athletic expressions [5]. One such novelty is electronic sports, or esports, which have emerged as a significant competitive phenomenon due to technological advancements over recent decades [6]. In the early 21st century, esports experienced a surge in popularity and an exponential increase in industry participants [7]. Major enterprises have established professional teams competing in international tournaments that attract millions of viewers worldwide [8,9]. Esports have transcended their niche origins, captivating a global audience and generating substantial financial rewards for top competitors. Games like Defense of the Ancients, League of Legends, and Counter-Strike: Global Offensive have become integral to modern culture [10]. The League of Legends World Championship finals in 2017 and 2018 exemplify esports’ extraordinary growth, with tickets selling out within seconds and the 2017 event drawing over 80 million viewers comparable to the 111 million viewers of the 2017 Super Bowl [11]. These events have become cultural milestones, fostering a sense of community among participants and spectators. The involvement of major sponsors, extensive media coverage, and significant prize pools have elevated esports to a status comparable to traditional sports, solidifying its legitimacy [12]. Technological advancements enable immersive gaming experiences, while online streaming platforms and social media facilitate widespread dissemination of events, nurturing a vibrant fan base. The accessibility of esports across computers, consoles, and mobile devices has broadened its appeal to a diverse audience. Esports offer unique opportunities for professionals and enthusiasts alike, becoming a lucrative industry with career prospects and economic growth potential. The competitive nature of esports cultivates valuable skills such as teamwork, communication, strategic thinking, and problem-solving, which are transferable to other life domains [13]. Recognizing this, educational institutions in several countries have integrated esports into curricula, fostering skills aligned with the digital era’s demands [14]. Moreover, research indicates that while esports cultivates valuable digital skills, it also impacts players’ social abilities, with findings showing esports players to be generally less extroverted and conscientious compared to traditional athletes [15]. Aspiring to become a professional e-athlete has become a coveted goal for many young individuals, capturing the imagination of a digitally native generation [16].

Gaming performance is influenced by various factors beyond technical specifications, notably cognitive functions (CFs) such as reaction time, eye–hand coordination, attention, working memory, and problem-solving abilities [17,18]. These CFs play a crucial role in gaming performance; notably, the cognitive demands necessary for peak performance can differ depending on the genre of the game. Studies have indicated that players of First-Person Shooter (FPS) games generally show quicker reaction times but reduced inhibitory control [19]. On the other hand, players of Multiplayer Online Battle Arena (MOBA) games frequently exhibit stronger working memory, sustained attention, and problem-solving abilities [20]. These findings underscore the importance of considering the specific cognitive demands of different game genres when designing interventions to optimize CFs in amateur e-athletes.

Evidence indicates that both conventional cognitive training and Virtual Reality (VR) interventions can induce significant improvements in CFs over short-term periods [21,22] as well as be a useful rehabilitation tool [23,24,25]. Similarly to VR, recent studies on brain stimulation techniques, such as the home-use of binaural beats, have shown complex, sometimes reverse effects on cognitive performance, emphasizing the need for in-depth, controlled intervention environments in cognitive en-hancement [26]. Specifically, VR training has been shown to enhance eye–hand coordination and reaction time [27], which are essential skills for gaming performance. Moreover, VR can prepare e-athletes for live events by simulating the atmosphere of a stadium or arena. This exposure helps reduce performance anxiety and ensures that players are equipped to handle the unique challenges associated with competing in front of large audiences [28]. The technological versatility of VR has rendered it a powerful tool not only for entertainment but also for various applications in training and therapy across multiple disciplines [29,30].

VR technology offers an innovative approach to training e-athletes by immersing them in dynamic and highly realistic digital environments [31]. This heightened level of immersion facilitates focused practice and skill refinement, providing e-athletes with unprecedented opportunities for targeted cognitive and skill development [32]. VR not only enhances technical abilities but also contributes to mental resilience by exposing players to high-pressure situations, enabling them to develop strategies for maintaining composure and focus during intense competitions [33]. What sets VR apart from traditional training methods is its ability to create interactive, three-dimensional virtual spaces that users can actively interact with [34]. Through sophisticated hardware and software, including head-mounted displays and motion tracking systems, VR simulates various sensory inputs such as visuals, sounds, and haptic feedback [35]. This immersive environment offers a compelling sense of realism and presence, allowing e-athletes to manipulate and navigate within the virtual world, performing actions that closely mimic real-world experiences.

To our knowledge, no studies have investigated VR-based cognitive stimulation specifically in e-athletes. Our research addresses this gap by evaluating the effectiveness of VR training on enhancing CFs critical to esports performance in amateur e-athletes. This study introduces a novel application of VR technology within the context of esports, potentially laying the foundation for future developments in esports training methodologies. By providing empirical evidence on the efficacy of VR-based cognitive training, we seek to contribute valuable insights that can enhance performance optimization strategies for e-athletes. The findings may have broader implications for the professionalization of esports and the development of standardized training protocols.

## 2. Methods

### 2.1. Participants

Based on earlier research regarding the influence of VR training on cognitive functions [36], the study included 66 participants (45 men and 21 women) ranging in age from 19 to 41 years, with a mean age of 23.96 ± 3.90 years. Participants were recruited through gaming communities, social media platforms, and among university students. Interested individuals were invited to complete a screening questionnaire to ensure they met the inclusion criteria for the study. Once eligibility was confirmed, participants were provided with detailed information about the study, and informed consent was obtained before they were officially enrolled. Using a simple 1:1 randomization process via the website https://www.randomizer.org/ (accessed on 12 December 2022), participants were randomly assigned to either the experimental group (E) (n = 32, 9 women and 23 men) or the control group (C) (n = 34, 12 women and 22 men). The experimental group underwent training for eight consecutive weekdays. The inclusion criteria specified that participants must be active amateur esports players, defined as those engaged in competitive, ranked matches, but without any prior participation in professional esports tournaments. To verify their status, participants were asked to provide their in-game identifiers, allowing us to confirm the accuracy of the data provided. Exclusion criteria included neurological, visual, auditory, or motor disorders that could impair their ability to participate in VR training. Additionally, participants who reported adverse side effects from virtual reality immersion were excluded following the random group assignment. Prior to the study, a questionnaire was administered to gather details about the participants’ most frequently played game types, daily gaming duration, esports experience, and age. Responses were similar across both groups, with MOBA games being the most common, followed by FPS games. No significant differences were observed between the experimental and control groups regarding average daily gaming time, esports experience, or age, as shown in Table 1 and determined by the Mann–Whitney U test. Additionally, initial reaction time, motor time, and eye–hand coordination showed no significant differences between groups, as confirmed by mixed model ANOVA comparisons.

To safeguard participants’ well-being, the Simulator Sickness Questionnaire (SSQ) [37] was used to monitor potential side effects from the VR training both before and after each session, with no participants reporting any symptoms. Additionally, SSQ use allowed us to monitor simulator usage, physical condition, health status, sleep, and alcohol consumption.

**Figure 1:**
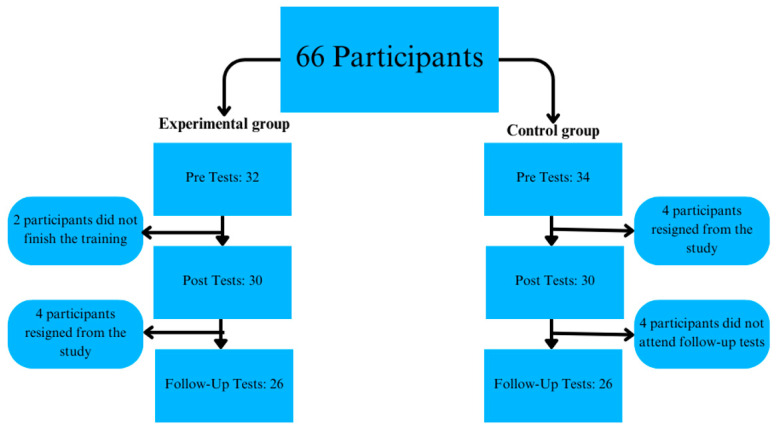
Distribution of participants during subsequent tests.

The study had a dropout rate of 21.22%, as shown in Figure 1, which details the distribution and retention of participants across subsequent testing sessions. Recruitment for the study took place in November and December of 2022, and data were collected in December 2022. The study adhered to the ethical standards of the Helsinki Declaration of 1964 and its subsequent revisions. The Research Ethics Committee of the Wroclaw University of Health and Sport Sciences approved the study’s methods and protocol (approval no. 19/2022), and all participants provided written informed consent prior to participation.

### 2.2. Measurements

**Figure 2:**
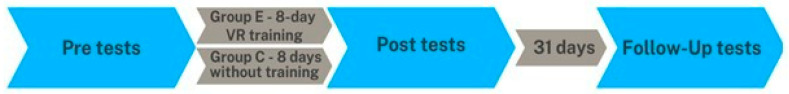
Progress chart for measurement protocol.

In the E group, CF assessments were conducted before the first training session and again 30 min after the final session. For the C group, pre- and post-tests were carried out with an eight-day gap between them, matching the timeframe of the E group. Additionally, follow-up tests were performed for both groups 31 days after the post-tests to evaluate any potential long-term effects of the training, with the measurement protocol outlined in Figure 2.

To assess eye–hand coordination (EHC), the S1 2HAND test from the Vienna Test System (VTS) (Mödling, Austria, 2013) was used [38]. In this task, participants maneuver two joysticks to guide a red dot along a track composed of three sections, each requiring different levels of coordination between the left and right hands. The test measured the total mean time to complete the task, reflecting overall performance, as well as the total percent error duration (TPED), which indicated the accuracy of EHC.

For reaction time (RT) and motor time (MT), the S2 Reaction Test also from the VTS was employed. In this test, participants responded to auditory cues by releasing a button and quickly pressing an adjacent one. The time between hearing the signal and releasing the button indicated RT, while the time taken to press the neighboring button measured MT. Both metrics were recorded in milliseconds.

### 2.3. Intervention

E-athletes assigned to the study group participants were subjected to a series of eight immersive VR training sessions in the game Beat Saber (https://beatsaber.com/, accessed on 10 December 2022), spanning eight consecutive weekdays from Tuesday to Thursday, with each session lasting 15 min. To provide the VR training environment, the Valve Index VR headset was utilized. On the other hand, participants assigned to the C group did not undergo any VR training. The primary objective of the Beat Saber game is to slice through colourful blocks that appear to the rhythm of the song, all while wielding two virtual lightsabers. Each block corresponds to a specific color, and players must strike the blocks with the lightsaber of the according color from the direction indicated by an arrow on each block. The game’s difficulty level progressively increased every two days, starting from the normal level and advancing to the expert level. The selection of songs played during the training sessions was randomized to ensure variation and prevent bias. This training approach in Beat Saber offers a dynamic and engaging experience for e-athletes, providing them with both cognitive and physical challenges. The rhythmic gameplay, combined with the need for quick reactions and precise eye–hand coordination, makes it an ideal platform for enhancing CFs. The randomized selection of songs further adds variability and adaptability to the training sessions, requiring players to quickly adapt to different rhythms and patterns, promoting cognitive flexibility and auditory processing skills.

### 2.4. Statistical Analysis

The statistical analyses for this study were performed at the Biostructure Laboratory of the Wroclaw University of Health and Sport Sciences, utilizing IBM SPSS Statistics 27 software (https://www.ibm.com/products/spss-statistics, accessed on 22 December 2022). The laboratory holds ISO 9001 [39] certification, ensuring compliance with rigorous quality standards. The statistical significance level was set at *p* < 0.05 to determine the presence of statistically significant findings. To assess the normality of the variables’ distribution, the Shapiro–Wilk test was employed.

Initially, mean values and standard deviations were calculated for the Reaction Test and 2HAND test results. A 2 × 3 × 6 mixed model ANOVA was then conducted, with the group (experimental vs. control) as the between-subjects factor, and measurement (RT, MT, EHC, TPED) and time (pre, post, follow-up) as within-subject factors. To identify significant differences between and within groups over time, a Bonferroni post-hoc test was applied. This method provided the foundation for the statistical analysis of the study.

## 3. Results

**Figure 3:**
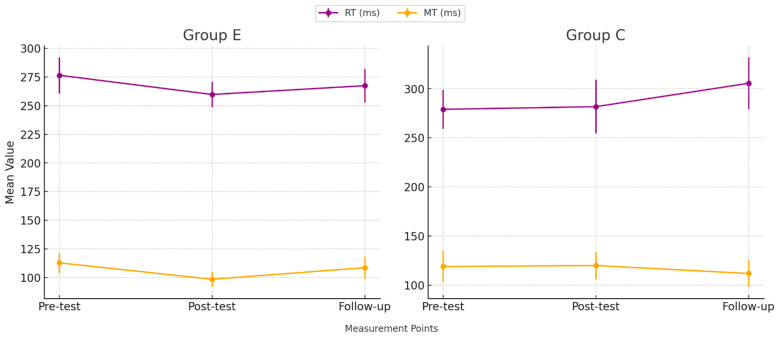
Results of the Reaction Test across three measurement points.

**Figure 4:**
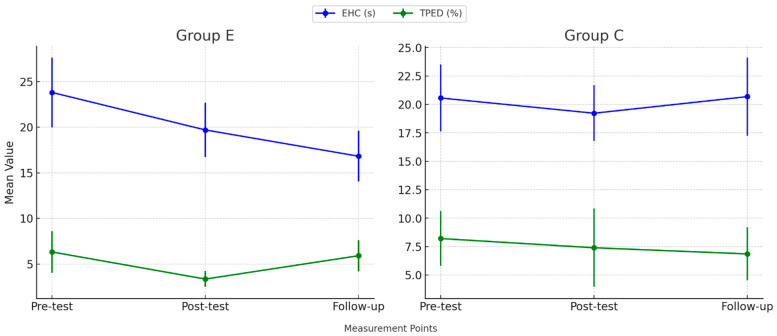
Results of 2HAND test across three measurement points.

The initial phase of our analysis focused on quantitatively evaluating the outcomes of the cognitive tests administered to the participants. Table 2 presents a comprehensive summary of these results, delineated as mean values ± standard deviations and additional percent change calculations between results obtained in each stage of testing, additionally Figure 3 and Figure 4 display CFs measurements taken across three points in time.

Table 3 presents the results from the mixed model ANOVA, highlighting the main effects and interactions. A significant main effect was found for Group (F = 6.363, *p* = 0.015, η^2^p = 0.113, λ = 6.363) and for Measurement (F = 2202.983, *p* < 0.001, η^2^p = 0.987, λ = 8811.931). However, no significant main effect was observed for Time (F = 1.686, *p* = 0.191, η^2^p = 0.033, λ = 3.372). Significant interactions were identified for Measurement × Group (F = 3.309, *p* = 0.012, η^2^p = 0.062, λ = 13.238) and the three-way interaction of Measurement × Time × Group (F = 2.333, *p* = 0.019, η^2^p = 0.045, λ = 18.661). However, the Time × Group interaction (F = 2.037, *p* = 0.136, η^2^p = 0.033, λ = 4.075) and Measurement × Time interaction (F = 1.602, *p* = 0.122, η^2^p = 0.031, λ = 12.818) did not reach statistical significance.

Following the initial analysis, significant within-group changes were investigated, as detailed in Table 4. For the E8 group, significant improvements were found in EHC (*p* < 0.001, F = 8.226, η^2^p = 0.251, λ = 16.453) and TPED (*p* = 0.004, F = 6.221, η^2^p = 0.203, λ = 12.442). However, no significant changes were observed in RT (*p* = 0.376, F = 0.998, η^2^p = 0.039, λ = 1.997) or MT (*p* = 0.103, F = 2.379, η^2^p = 0.089, λ = 4.759). Conversely, the C groups did not exhibit significant changes in measured CFs.

**Figure 5:**
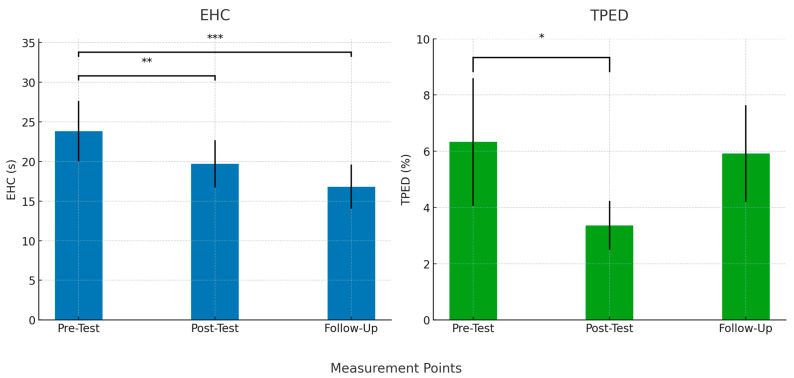
Significant within-groups comparisons. Note: * Significant at *p* < 0.05, ** Significant at *p* < 0.01, *** Significant at *p* < 0.001.

Subsequent Bonferroni post-hoc within-group analysis, as shown in Figure 5 and Table 5, indicated that the E group experienced significant improvements after the intervention, with some improvements also observed in pre- vs. follow-up comparisons. Specifically, in E, the comparison between pre-test and post-test revealed significant differences in EHC (*p* = 0.004) and TPED (*p* = 0.014). However, MT (*p* = 0.098) and RT (*p* = 0.479) did not show significant changes. Between pre-test and follow-up, only EHC remained significant (*p* < 0.001), while RT (*p* = 1.0), MT (*p* = 1.0), and TPED (*p* = 1.0) showed no significant differences. Post-test vs. follow-up comparisons showed no significant changes in RT (*p* = 1.0), MT (*p* = 0.476), or EHC (*p* = 0.153), while TPED approached significance (*p* = 0.065).

For the C, there were no significant changes between pre- and post-tests (RT, *p* = 1.0; MT, *p* = 1.0; EHC, *p* = 0.813; TPED, *p* = 1.0). Similarly, comparisons between pre-test and follow-up were non-significant (RT, *p* = 0.126; MT, *p* = 0.748; EHC, *p* = 1.0; TPED, *p* = 0.992). Additionally, post-test vs. follow-up comparisons yielded no significant changes for any metric (RT, *p* = 0.147; MT, *p* = 0.971; EHC, *p* = 0.968; TPED, *p* = 1.0).

## 4. Discussion

The objective of this study was to evaluate the effectiveness of immersive VR training in improving reaction time, motor time, and eye–hand coordination. VR environments offer customizable challenges that are difficult to reproduce in conventional cognitive training programs.

The results showed significant improvements in EHC and TPED, but there were no significant changes in reaction time or motor time. This outcome contrasts with the findings of Rutkowski et al. [40], who reported improvements in both EHC and reaction time among young musicians after five VR training sessions. The difference in results may be due to varying baseline levels of cognitive functions between study participants. E-athletes, as previous research has shown [19], tend to have faster reaction times, possibly giving them a cognitive advantage that may explain the lack of further improvement. Unfortunately, no follow-up tests were conducted in this study. The authors of the mentioned research suggest that the immersive, interactive nature of the VR system, combined with its ability to provide personalized feedback, may have contributed to the observed improvements in motor skills. Beat Saber not only served as a source of entertainment or cognitive training but also as a rehabilitation tool. A study conducted [41] in clinical groups highlights the potential of immersive VR games like Beat Saber also as a complementary therapy option for individuals with chronic stroke. The rhythmic gameplay, combined with the visual and auditory stimuli, encouraged patients to actively participate in rehabilitative exercises and engage in repetitive movements that can help restore motor function and improve CFs.

The improvements observed in our study may be explained by the engagement of multiple sensory modalities during VR training, including vision, hearing, and touch. Multisensory stimulation in VR enhances brain plasticity by driving changes in key regions such as the parietal and occipital cortices, which are involved in visuospatial processing [42]. Studies demonstrate that immersive VR experiences increase functional connectivity between the frontal and occipital regions, supporting improvements in visuospatial and executive functions [43], which development can be crucial not only in esports but also in highly specified real-life scenarios [44]. Additionally, VR training enhances visuomotor coordination by modulating neural circuits involved in motor and cognitive tasks [45], and this repeated stimulation improves visuospatial skills through greater neural efficiency and plasticity [46]. VR-based cognitive training has been shown to improve visuospatial memory and cognitive flexibility in various populations, including those at risk for cognitive decline [47,48]. In addition, studies report that VR can modulate cognitive function by improving sensory integration, which supports both motor learning and memory consolidation [49] and enhances overall neural efficiency, especially in older adults [50]. Our results align with those of Calabrò et al. [51], who observed similar improvements in eye–hand coordination following a VR intervention. Their study showed increased cortical activation in the fronto-parieto-occipital regions during VR motor training, which suggests that our VR-based motor training may have induced structural changes in the brain, particularly within the primary motor cortex. The ability of VR training to stimulate neuroplasticity in critical motor areas further underscores its potential as an effective tool for enhancing motor functions.

However, the specific neural mechanisms underlying these cognitive improvements remain unclear, and deeper research is needed to fully understand the role of VR in driving neuroplasticity [52,53]. Our study did not measure neural changes directly, and we can only rely on these findings as a theoretical framework to explain our results. While previous studies suggest potential structural changes in the brain due to VR training [54], our research cannot confirm these neural adaptations, underscoring the need for further investigation into the neural factors contributing to the cognitive benefits observed with VR interventions.

Another key factor contributing to the success of VR training is the physical activity [55] involvedin games like Beat Saber, which can be qualified as moderate [40]. Physical activity has been shown to reduce stress levels, which is crucial since chronic stress can impair CFs such as reaction time and eye–hand coordination [56]. By mitigating stress, moderate physical activity allows the brain to perform these tasks more efficiently, leading to improvements in both motor and cognitive performance. Beyond stress reduction, moderate physical activity promotes overall brain health by increasing blood flow and oxygen to the brain, supporting neurogenesis, and improving cognitive flexibility and processing speed [57]. Physical activity also triggers the release of neurotransmitters like dopamine and serotonin, which play important roles in enhancing mood, motivation, and CFs. Dopamine, in particular, is closely tied to reward systems and learning, while serotonin contributes to emotional regulation and cognitive processes such as memory consolidation [58]. Additionally, the immediate feedback and rewards in VR environments further stimulate the brain’s reward pathways, reinforcing learning and task engagement [59].

Our findings partially align with existing literature, demonstrating that immersive VR training can effectively improve specific CFs. However, the lack of significant improvement in reaction time and motor time suggests that the benefits of VR training may vary depending on the targeted CFs and participant characteristics. The improvements observed in our study support the notion that VR environments, with their capacity to engage in multiple sensory modalities and integrate moderate physical activity, hold promise for enhancing cognitive and motor skills. While our results reinforce the effectiveness of VR-based interventions, further research is needed to explore the variability in outcomes and to optimize the design of VR training protocols for a wider range of cognitive and motor functions.

## 5. Conclusions

The aim of this study was to assess the efficacy of immersive VR training in enhancing CFs. The findings revealed that the VR training conducted in Beat Saber resulted in a significant improvement in EHC and total TPED. Importantly, the cognitive gains in EHC were sustained over time, indicating the lasting impact of the training. The immersive eight-day VR training demonstrated its potential to enhance key CFs in amateur e-athletes, making it a valuable resource for foundational esports training. This approach could support the development of skills essential for esports competitiveness. However, no significant differences were found in RT and MT after eight weeks of training, which suggests that the effects of VR training may be more pronounced in specific cognitive domains.

While these results are promising, it is important to recognize that the direct impact of such training on actual esports performance remains to be fully explored. Future research should investigate whether the improvements in CFs translate into measurable gains in competitive gaming scenarios. Additionally, given the lack of observed changes in RT and MT, subsequent studies could explore longer training durations or varying intensities to determine optimal training protocols for these specific CFs. However, in the case of extended training periods or increased intensity, it is crucial to prioritize testing for potential side effects, such as simulator sickness, to ensure the safety and well-being of participants during VR interventions.

Moreover, the findings from this study can serve as a catalyst for further research into the integration of VR cognitive training not only in skill development but also in rehabilitation settings. Exploring the application of VR in therapeutic contexts could provide insights into its effectiveness for cognitive rehabilitation following neurological impairments. By expanding the scope of VR training research, it is possible to better understand its full potential and establish comprehensive guidelines for its application across different domains.

## 6. Limitations

Despite our rigorous efforts to conduct a comprehensive study, certain limitations need to be acknowledged. One was the inability to supervise our participants’ activities on Saturdays and Sundays. While we provided clear instructions for them to refrain from altering their current lifestyle, we were unable to monitor their adherence to these instructions outside the research environment. Another limitation pertains to the self-report nature of questionnaires. Self-report measures may be susceptible to response biases, such as social desirability or recall biases. Participants might have been inclined to present themselves in a favorable light, leading to potential inaccuracies in the data. Additionally, participant dropouts presented another limitation, which may have affected the study’s statistical power and the generalizability of the results. Dropouts could lead to a reduction in sample size, possibly limiting the strength of our findings or creating a biased representation of the population if those who completed the study differed systematically from those who withdrew. In future studies, strategies such as maintaining offering incentives or providing more flexible scheduling could help mitigate dropout rates.

## Figures and Tables

**Table 1:** Mean values ± SD of participants’ age and other questionnaire items.

Variable	E8	C8	Between-Groups Mann-Whitney U Test *p*-Values
Age	23.96 ± 3.90	23.79 ± 2.67	0.974
Average Daily gaming time	2.38 ± 1.43	2.35 ± 1.63	0.719
Years of Esports experience	3.58 ± 2.83	2.85 ± 1.70	0.429

**Table 2:** Test results presented as mean values ± standard deviation with Δ%.

Group	Variable	Pre Test Mean ± SD	Post Test Mean ± SD	Δ% Pre- Post	Follow-Up Test Mean ± SD	Δ% Pre- Follow-Up	Δ% Post- Follow-Up
E	RT (ms)	276.54 ± 15.84	259.77 ± 11.13	−6.06%	267.46 ± 14.68	−3.27%	2.96%
	MT (ms)	112.85 ± 8.80	98.5 ± 6.74	−12.71%	108.65 ± 9.82	−3.70%	10.29%
	EHC (s)	23.81 ± 3.83	19.70 ± 2.99	−17.25%	16.82 ± 2.78	−29.38%	−14.58%
	TPED (%)	6.33 ± 2.27	3.36 ± 0.87	−46.92%	5.92 ± 1.72	−6.47%	76.19%
C	RT (ms)	279.04 ± 19.84	281.77 ± 27.46	0.97%	305.54 ± 26.67	9.52%	8.44%
	MT (ms)	119 ± 15.73	120 ± 13.84	0.84%	111.88 ± 13.75	−6.01%	−6.93%
	EHC (s)	20.57 ± 2.93	19.23 ± 2.44	−6.51%	20.69 ± 3.44	0.58%	7.60%
	TPED (%)	8.21 ± 2.40	7.40 ± 3.45	−9.86%	6.86 ± 2.33	−16.45%	−7.30%

Note: RT—Reaction Time, MT—Motor Time, EHC—Eye–Hand Coordination, TPED—Total Percent Error Duration.

**Table 3:** Results of mixed model ANOVA for main effects and interactions.

Factor/Interaction	F-Value	df	*p*-Value	η^2^p	λ
Group	6.363	1	0.015 *	0.113	6.363
Measurement	2202.983	4	<0.001 ***	0.987	8811.931
Time	1.686	2	0.191	0.033	3.372
Time × Group	2.037	2	0.136	0.033	4.075
Measurement × Group	3.309	4	0.012 *	0.062	13.238
Measurement × Time	1.602	8	0.122	0.031	12.818
Measurement × Time × Group	2.333	8	0.019 *	0.045	18.661

Note: * Significant at *p* < 0.05, *** Significant at *p* < 0.001.

**Table 4:** Results of mixed model ANOVA for within-groups changes.

Group	Variable	*p*-Values	F-Value	η^2^p	λ
E8	RT (ms)	0.376	0.998	0.039	1.997
	MT (ms)	0.103	2.379	0.089	4.759
	EHC (s)	<0.001 ***	8.226	0.251	16.453
	TPED (%)	0.004 **	6.221	0.203	12.442
C8	RT (ms)	0.079	2.681	0.099	5.361
	MT (ms)	0.445	0.823	0.033	1.647
	EHC (s)	0.368	1.021	0.040	2.043
	TPED (%)	0.609	0.500	0.020	1.001

Note: RT—Reaction Time, MT—Motor Time, EHC—Eye–Hand Coordination, TPED—Total Percent Error Duration. ** Significant at *p* < 0.01, *** Significant at *p* < 0.001.

**Table 5:** Results of Bonferroni post-hoc within-groups comparisons between measurement points presented as *p*-values.

Group	RT			MT			EHC			TPED		
	Pre vs. Post	Pre vs. Follow-Up	Post vs. Follow-Up	Pre vs. Post	Pre vs. Follow-Up	Post vs. Follow-Up	Pre vs. Post	Pre vs. Follow-Up	Post vs. Follow-Up	Pre vs. Post	Pre vs. Follow-Up	Post vs. Follow-Up
E8	0.479	1.0	1.0	0.098	1.0	0.476	0.004 **	<0.001 ***	0.153	0.014 *	1.0	0.065
C8	1.0	0.126	0.147	1.0	0.748	0.971	0.813	1.0	0.968	1.0	0.992	1.0

Note: RT—Reaction Time, MT—Motor Time, EHC—Eye–Hand Coordination, TPED—Total Percent Error Duration. * Significant at *p* < 0.05, ** Significant at *p* < 0.01, *** Significant at *p* < 0.001.

## Data Availability

Raw data presented in the study are not publicly available to preserve individual privacy under the European General Data Protection Regulation. To access the data, the first author should be contacted.
